# Establishment and Characterization of Primary Cultures from Iranian Oral Squamous Cell Carcinoma Patients by Enzymatic Method and Explant Culture

**Published:** 2017-07

**Authors:** Meysam Ganjibakhsh, Pouyan Aminishakib, Parvaneh Farzaneh, Abbas Karimi, Seyed Abolhassan Shahzadeh Fazeli, Moones Rajabi, Ahmad Nasimian, Fereshteh Baghai Naini, Hedieh Rahmati, Neda Sadat Gohari, Nazanin Mohebali, Masoumeh Asadi, Zahra Elyasi Gorji, Mehrnaz Izadpanah, Shiva Mohamadi Moghanjoghi, Sepideh Ashouri

**Affiliations:** 1 PhD Student, Human and Animal Cell Bank, Iranian Biological Resource Center, ACECR, Tehran, Iran; Department of Anatomy, School of Medicine, Iran University of Medical Sciences, Tehran, Iran; 2 Assistant Professor, Dental Research Center, Dentistry Research Institute, Tehran University of Medical Sciences, Tehran, Iran; Department of Oral and Maxillofacial Pathology, School of Dentistry, Tehran University of Medical Sciences, Tehran, Iran; 3 Assistant Professor, Human and Animal Cell Bank, Iranian Biological Resource Center, ACECR, Tehran, Iran; 4 Assistant Professor, Craniomaxillofacial Research Center, Dentistry Research Institute, Tehran University of Medical Sciences, Tehran, Iran; Department of Oral and Maxillofacial Surgery, School of Dentistry, Tehran University of Medical Sciences, Tehran, Iran; 5 Associate Professor, Human and Animal Cell Bank, Iranian Biological Resource Center, ACECR, Tehran, Iran; Department of Molecular and Cellular Biology, School of Basic Science and Advanced Technologies in Biology, University of Sciences and Culture, Tehran, Iran; 6 Adjunct Assistant Professor, Department of Oral and Maxillofacial Pathology, School of Dentistry, Tehran University of Medical Sciences, Tehran, Iran; 7 PhD Student, Human and Animal Cell Bank, Iranian Biological Resource Center, ACECR, Tehran, Iran; Department of Clinical Biochemistry, School of Medical Sciences, Tarbiat Modares University, Tehran, Iran; 8 Associate Professor, Dental Research Center, Dentistry Research Institute, Tehran University of Medical Sciences, Tehran, Iran; Department of Oral and Maxillofacial Pathology, School of Dentistry, Tehran University of Medical Sciences, Tehran, Iran; 9 Researcher, Human and Animal Cell Bank, Iranian Biological Resource Center, ACECR, Tehran, Iran; 10 PhD Student, Department of Applied Cell Science and Tissue Engineering, School of Advanced Technologies in Medicine, Tehran University of Medical Sciences, Tehran, Iran

**Keywords:** Carcinoma, Squamous Cell of Head and Neck, Primary Cell Culture, Mouth Neoplasms

## Abstract

**Objectives:**

Oral Squamous Cell Carcinoma (OSCC) is the most frequent oral cancer worldwide. It is known as the eighth most common cancer in men and as the fifth most common cancer in women. Cytogenetic and biochemical studies in recent decades have emphasized the necessity of providing an appropriate tool for such researches. Cancer cell culture is a useful tool for investigations on biochemical, genetic, molecular and immunological characteristics of different cancers, including oral cancer. Here, we explain the establishment process of five primary oral cancer cells derived from an Iranian population.

**Materials and Methods:**

The specimens were obtained from five oral cancer patients. Enzymatic, explant culture and magnetic-activated cell sorting (MACS) methods were used for cell isolation. After quality control tests, characterization and authentication of primary oral cancer cells were performed by short tandem repeats (STR) profiling, chromosome analysis, species identification, and monitoring the growth, morphology and the expression of CD326 and CD133 markers.

**Results:**

Five primary oral cancer cells were established from an Iranian population. The flow cytometry results showed that the isolated cells were positive for CD326 and CD133 markers. Furthermore, the cells were free from mycoplasma, bacterial and fungal contamination. No misidentified or cross-contaminated cells were detected by STR analysis.

**Conclusions:**

Human primary oral cancer cells provide an extremely useful platform for studying carcinogenesis pathways of oral cancer in Iranian population. They may be helpful in explaining the ethnic differences in cancer biology and the individuality in anticancer drug response in future studies.

## INTRODUCTION

Oral squamous cell carcinoma (OSCC) is the most common oral cancer worldwide. It is known as the eighth most common cancer in men and as the fifth most frequent cancer in women [1]. Various prevalence rates have been reported throughout the world for OSCC, depending on different geographical regions [2]. The prevalence of head and neck SCC (HNSCC) in Iran is approximately similar to that in other Asian countries, such as India and Pakistan [3]. Despite recent advances in the treatment of OSCC, survival rates of the patients have not significantly improved [1]. The 5-year survival rate of the OSCC patients, younger than 45 years old, has been reported to be 61% [4]. The first human cancer cell line was cultured in 1951, established from cervical carcinoma tissue [5]. This cell line provided a reliable material for researchers to study the cancer without the common problems usually encountered upon using non-vital cancer cells [6]. Furthermore, the most recent attempts are focused on characterizing and isolating the key cells responsible for self-renewal and progression of tumors, known as “Cancer Stem Cells” [7]. So far, over three hundred HNSCC cell lines have been established worldwide. Viable HNSCC cells, derived from fresh cancer specimens, have also been recently identified as a valuable tool for investigations on molecular mechanisms and genetic alterations involved in initiation and progression of this cancer [8]. In this study, we successfully established a total number of five human primary oral cancer cells from an Iranian population. The isolated cells will provide an extremely useful platform for studying carcinogenesis pathways of oral cancer in the Iranian population. The primary cells will be helpful in understanding the ethnic differences in cancer biology and individuality in anticancer drug response in future studies. These cells are extremely valuable, since the primary culture imitates the in-vivo conditions. As it may be difficult to understand the genetic and epigenetic varieties of millions of patients from a small number of cancer cell lines, the primary cell culture serves as a potential tool to improve the efficiency of conventional clinical approaches to treat cancer and develop successful personalized medicine.

## MATERIALS AND METHODS

Materials that have been used in this study include: Dulbecco’s Modified Eagle’s Medium (DMEM, Invitrogen, Massachusetts, USA), Ham’s F12 Medium (Invitrogen, Massachusetts, USA), Fetal bovine serum (FBS) (Invitrogen, Massachusetts, USA), Penicillin-Streptomycin (Sigma Aldrich, Missouri, USA), Potassium dihydrogen phosphate (Merck, Darmstadt, Germany), Sodium phosphate dibasic (Sigma Aldrich, Missouri, USA), Minimum Essential Medium (MEM, Invitrogen, Massachusetts, USA), Epithelial growth factor (EGF, Royan, Tehran, Iran), Hydrocortisone (Sigma-Aldrich, Missouri, USA), Cholera toxin (Sigma-Aldrich, Missouri, USA), Sodium chloride (Merck, Darmstadt, Germany), Potassium chloride (Merck, Darmstadt, Germany), L-Glutamine (Invitrogen, Massachusetts, USA), Trypsinethylenediaminetetraacetic acid (EDTA) (Invitrogen, Massachusetts, USA), Trypan blue (Sigma Aldrich, Missouri, USA), Dimethyl sulfoxide (DMSO, Sigma-Aldrich, Missouri, USA), Thioglycollate Broth (Merck, Darmstadt, Germany), Tryptone Soy Broth (Sigma-Aldrich, Missouri, USA), Collagenase type I (Sigma Chemical Co., Munich, Germany), PPLO Broth (BD, New Jersey, USA), PPLO Agar (BD, New Jersey, USA), Colchicine (Sigma-Aldrich, Missouri, USA), Methanol (Sigma-Aldrich, Missouri, USA), Acetic acid (Sigma-Aldrich, Missouri, USA), Polyethylenimine (Sigma-Aldrich, Missouri, USA), Dako flow cytometry system (Partec, Germany), CD marker and isotype matched controls (Serotec, London, UK) and MACS kit (Miltenyi Biotec, USA).

### Collection of samples:

Tissue samples were obtained from five oral cancer patients, who underwent surgery at two referral hospitals of Tehran University of Medical Sciences, with prior informed consent (Tehran University of Medical Sciences Ethical Approval Committee # IR.TUMS.REC.1394.1285). Table 1 shows the essential information regarding the samples.

**Table 1.** Information related to the cell lines obtained from cancerous oral tissues

	**Cell name**	**Accession number**	**Gender**	**Age at sampling time**	**Cancer location**	**Pathologic diagnosis**	**Doubling time (h)**	**Cell growth characteristics**	**CD 326 expression (%)**	**CD 133 expression (%)**
1	OCC-22	IBRC C11066	Female	82	Palatal mucosa	Well differentiated	27.1	Adherent	52.58	24.04
2	OCC-25	IBRC C11069	Female	57	Tongue	Moderately differentiated	26.9	Adherent	60.88	45.83
3	OCC-31	IBRC C11075	Male	65	Tongue	Well differentiated	25.8	Adherent	64.87	45.4
4	OCC-32	IBRC C11076	Female	55	Buccal mucosa	Moderately differentiated	26.5	Adherent	69.03	51.32
5	OCC-34	IBRC C11078	Male	60	Buccal mucosa	Well differentiated	26.3	Adherent	62.55	41.44

### Cell isolation with explant culture method:

Oral cancer tissue samples were immersed in 70% ethanol for 20 seconds, and after that, they were transferred to phosphate buffered saline (PBS). The samples were washed several times with PBS, and then, they were transferred to DMEM. After removing the blood vessels and debris from the samples, they were cut into smaller segments, and each segment was divided into approximately 1mm^3^ pieces. Every two or three small tissue pieces were seeded in 35-mm^2^ tissue culture dishes covered with a sterile 22-mm^2^ glass slip. The complete culture media included: DMEM: Ham’s F12 medium (1:1), 10% FBS, 100U/ml penicillin, 100μg/ml streptomycin, 6mM L-glutamine, 10μg/ml insulin, 20ng/ml EGF, 0.5μg/ml hydrocortisone, 100ng/ml cholera toxin and 2mM MEM, which were added to each cell culture plate. The primary cell cultures were incubated at 37°C with 5% CO_2_ for approximately three weeks to reach the 70–80% confluence. The cell cultures were observed daily, and the medium was changed every 3 or 4 days. When the cells reached the 70–80% confluence, the plates were subcultured by using 0.25% trypsin and 0.02% EDTA solution and were spared into culture flasks containing complete culture media at a seeding density of 4×10^4^ cells/cm^2^. The primary cells were incubated at 37°C with 5% CO_2_ to reach the 80% confluence.

### Cell isolation with the enzymatic method:

In enzymatic digestion process, the samples were initially washed thoroughly with sterile PBS containing penicillin (500U/ml) and streptomycin (500μg/ml). Then, the samples were cut into 1mm^3^ pieces, followed by incubation with 0.1% collagenase type I at 37 °C for 60 minutes, while shaken vigorously to separate oral cancer cells. For neutralizing the collagenase activity, the DMEM containing 10% FBS was added. To remove debris, the dissociated tissues were filtered through 100, 70 and 40 μm cell strainers followed by centrifugation at 300 ×g for 10 minutes. In order to remove the contaminating erythrocytes, the samples were incubated at room temperature for 10 minutes in lysis buffer (155mM NH_4_Cl, 5.7mM K_2_HPO_4_ and 0.1mM EDTA, pH=7.3). Afterwards, the cells were centrifuged at 300 ×g for 5 minutes and were incubated at 37°C with 5% CO_2_ in the complete culture medium for approximately two weeks. The medium was changed every 3 or 4 days. After the cells reached the 70–80% confluence, trypsin-EDTA was used to subculture the cells into the culture flasks containing complete culture media, at a seeding density of 4×10^4^ cells/cm^2^.

### Magnetic-activated cell sorting (MACS):

With the aim of epithelial cancer cell enrichment, positive selection MACS with CD326 Microbeads was used according to the manufacturer’s instructions. For this purpose, the primary cells with 80% confluence were trypsinized and resuspended in 100μl of MACS separation buffer. The cells were incubated with 10μl of CD326 Epithelial cell adhesion molecule (EpCAM) MicroBeads, according to the manufacturer’s instructions. Next, the magnetically labeled CD326+ cells were isolated by using a magnetic column. Finally, the CD326+ cells were cultured in complete culture medium.

### Cell cryopreservation:

The cell cultures were maintained in lag phase to reach optimal growth rate and good cell recovery. The fresh growth medium was added to the cell culture, 24 hours prior to freezing. The cell count and viability were assessed by trypan blue staining [1]. The cell pellet was harvested and then resuspended in freezing medium containing 90% FBS and 10% DMSO, to reach a final cell density of 1–2×10^6^ viable cells/ml. The cell suspension was transferred into sterile cryovials labeled with cell name, Iranian Biological Research Center (IBRC) ID number, date of freeze and cell count. The cryovials were stored at −20°C for one hour, and afterward, at −80°C for one day and then, they were transferred to −196°C nitrogen tank. The cells were further checked for viability and contamination at the intervals of two weeks, six months and one year after freezing.

### Detection of fungal and bacterial contamination:

During the culture process, the cells were routinely investigated under the microscope for fungal and bacterial contamination. For further confirmation, the cell supernatant (antibiotic-free) was separately cultured in thioglycollate broth and tryptone soy broth media for 14 days at 22°C and 32°C.

### Mycoplasma detection:

Mycoplasma contamination was checked using mycoplasma PCR detection kit (IBRC, C1111) and direct solid agar microbiological culture. PCR method can detect the most common mycoplasma species in the cell culture including *M. orale, M. hyorhinis, M. arginini, M. fermentans, M. hominis, M. bovis, and A. laidlawii* [2]. To confirm the Multiplex PCR analysis, the supernatant of the cultured cells was inoculated into PPLO broth and PPLO agar, supplemented with nutritive enrichments. The culture was incubated at 32°C for at least three weeks before mycoplasma testing.

### Species identification:

Genomic DNA was extracted from the primary cells via column-based DNA extraction kit (IBRC: MBK0021). The authentication of the primary cell was confirmed by amplification of cytochrome C oxidase subunit I (COI) mitochondrial gene using Multiplex PCR method. The specific primers were used as reported by Cooper et al [9]. Fourteen species including mouse, rat, rabbit, camel, horse, cow, sheep, cat, dog, guinea pig, pig, rhesus monkey, African green monkey, Chinese hamster, chicken, and human can be detected by this method.

### Growth curve:

Approximately 5×10^4^ cells/ml were seeded into 24-well plates and were cultured for 6 days. The cell concentration and growth rate were recorded triplicate every day. After that, the cell growth curves and the population doubling time were determined.

### Chromosome analysis:

After the cells reached the 50–60% confluence, Colcemid was added to the medium with a final concentration of 20 μl/ml, followed by incubation at 37°C for 0.5–1 hour. The medium was then removed and washed with PBS. The cells were trypsinized and centrifuged at 300 ×g for 5 minutes. After removing the supernatant, the hypotonic solution (0.075M KCl) was added to the cell pellet and incubated for 40 minutes at 37°C. Next, 1ml of cold fixative (3:1 methanol and acetic acid) was added and centrifuged for 10 minutes at 300 ×g. The cell pellet was resuspended in 5ml of cold fixative and was centrifuged for 5 minutes at 300 ×g. Finally, the fixative was discarded, and the pellet was resuspended in 1ml of fresh fixative. The suspension was placed on slides and was dried at 65°C for 18 hours. The slides were then placed in 0.025 % trypsin solution for 35 seconds. The solution was removed and the slides were exposed to PBS and were stained with Giemsa for 5 minutes. The slides were then rinsed in distilled water and were air-dried. At least 30 to 50 metaphases were scored and analyzed.

### Cell authentication by short tandem repeats (STR) analysis:

In order to confirm the absence of cross-contamination between the cells, STR profiling was performed separately for each sample. This technique is one of the few DNA profiling technologies available for routine identification (authentication) of human cell lines, stem cells and tissues. The Iranian Biological Resource Center (IBRC) has conducted STR method with 16 markers from Applied Biosystems (AmpFlSTR® Identifiler® Plus PCR Amplification Kit, Cat# 4440211) for authentication of human cells.

### Flow cytometry analysis:

Immunophenotyping of oral cancer cells was performed by direct immunofluorescence staining of cell surface antigens using FITC or RPE conjugated antibodies against CD326, CD133, and appropriate isotype-matched controls. The samples were analyzed at the fifth passage in Dako flow cytometry system, using the FlowMax software.

## RESULTS

### Morphological observation of the primary cells:

**Fig. 1:**
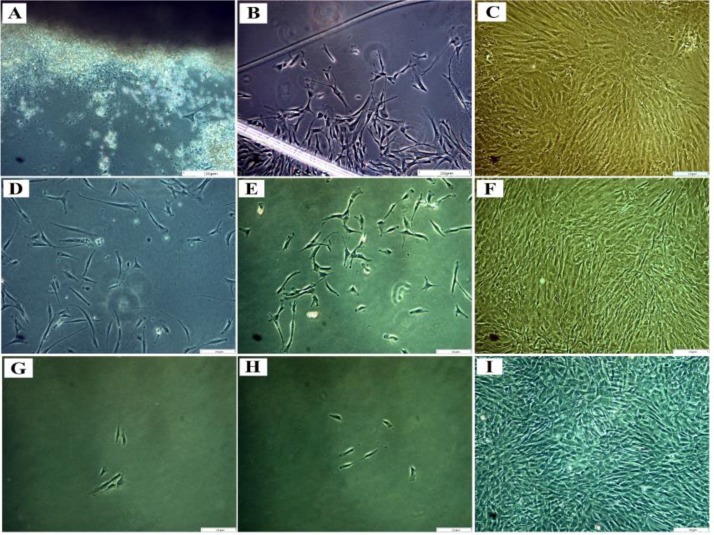
Images A, B and C show cells obtained by the explant method. (A) After 48 hours. (B) After 10 days. (C) After 3 weeks. In D, E and F, the cells were isolated using the enzymatic method. (D) After 24 hours. (E) After 48 hours. (F) After 10 days. The CD326 (EpCAM) positive cells, which were isolated using the magnetic-activated cell sorting (MACS) method were pictured after 24 hours (G, H), and after 10 days (I)

Enzymatic and explant methods were used in this study. In the explant method, the cells were outgrowing from tissue pieces two days after being plated in the culture flask, while in the enzymatic method, the cells were observed 12 hours after the primary culture. In both methods, morphological observation showed both epithelial and fibroblast cells in the cell culture. Thus, in comparison to the explant technique, the enzymatic method represented higher efficiency and was less time-consuming. It should be noted that the number of the cells obtained at the beginning of the primary culture in enzymatic digestion was more than that in the explant method. Nevertheless, different isolation methods do not affect the morphological and phenotypic characteristics of cells (Fig. 1).

### Microbial analysis:

**Fig. 2:**
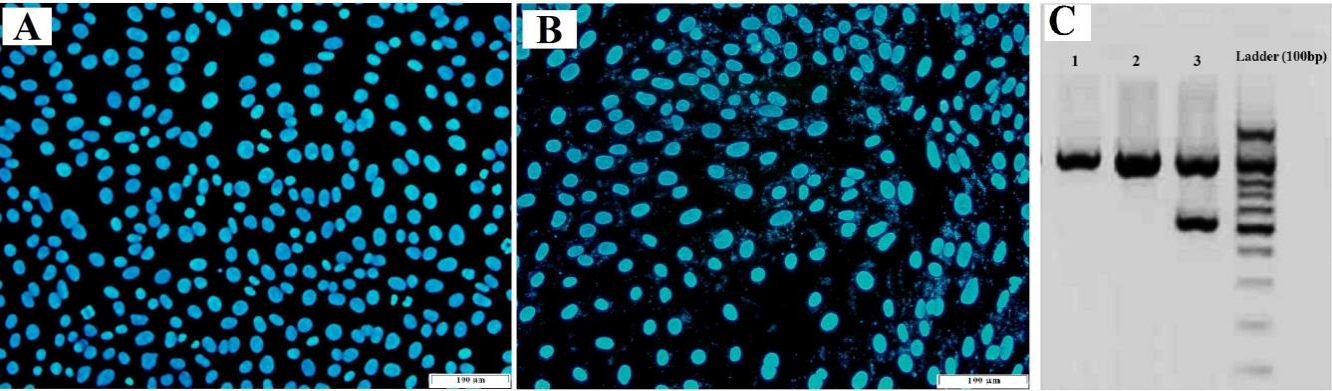
(A) Primary oral squamous carcinoma cells stained with Hoechst 33258. (B) Positive control. (C) PCR analysis of mycoplasma contamination

Based on the obtained results, the culture medium remained clear and did not display any visible changes; whereas, the positive test control was visibly turbid with precipitation. These results demonstrated that the newly-established human primary oral cancer cells were not contaminated by bacteria or fungi. The results of fluorescence microscopy by Hoechst 33258 showed that the human primary oral cancer cells were free from mycoplasmas. After being stained with Hoechst 33258, the cells displayed smooth surfaces and round nuclei with blue fluorescence, and no filamentous blue fluorescence was detected nearby the nuclei (Fig. 2. A–B).

Presence of punctiform and filiform blue fluorescence in the cell nucleoli could indicate mycoplasma contamination [**10**]. These results were further confirmed by mycoplasma PCR detection method (Fig. 2. C).

### Chromosome analysis:

**Fig. 3:**
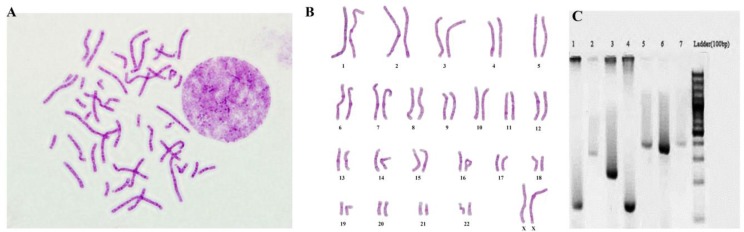
(A) Metaphase spread and (B) Karyotype of oral squamous cell carcinoma (OSCC), which indicate the absence of numerical abnormality in the majority of the cells. (C) Identification of species by multiplex PCR using specific primers. Lane 5: human primary oral cancer cells with no cross-contamination between this cell line and other species. The controls, lane 1 and 4: mouse samples (NIH3T3 and L-929 cell lines), lane 2: hamster sample (CHO cell line), lane 3: chicken sample (CHI05), lane 6: cat sample and lane 7: human sample (A549 cell line)

The spreads of metaphases at passages 5 to 10 were analyzed. The chromosome numbers of the primary oral cancer cells were counted 2n = 46 in the majority of the cells, indicating there is no numerical chromosomal abnormality in these cells (Fig. 3. A–B).

### Species identification:

The PCR amplification for the established human primary oral cancer cells yielded a 391bp fragment in each primary oral cancer cell, which is associated with human species. The control groups for other species such as mouse (150-bp), rat (196-bp), cat (340-bp), hamster (315-bp) and chicken (550-bp) had different specific bands. These results confirmed the cell delineation of the species and also revealed no cross-contamination with other species (Fig. 3. C).

### Growth curve:

**Fig. 4:**
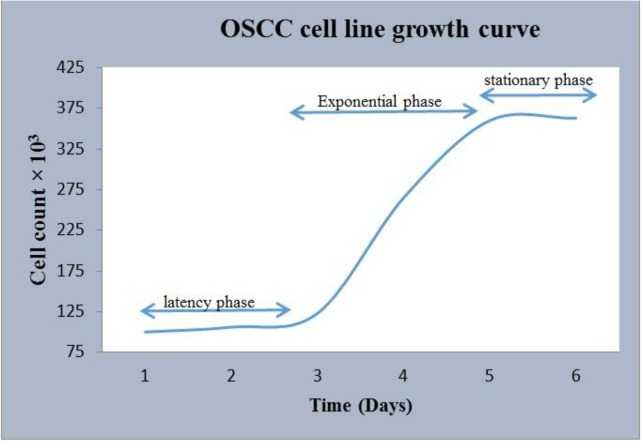
Growth curve of the primary oral cancer cells. The growth curve has the typical S-shaped pattern, and it is divided into three sections including a latency phase, an exponential phase, and a stationary phase

The growth curve diagram for the primary oral cancer cells indicated a sigmoid growth pattern. Because of the protease damage following the use of trypsin, the oral cancer cells stayed in lag phase during the first three days in culture, and subsequently, the lag phase reappeared after day 5. Finally, the cells entered the stationary phase after another 48 hours, which established the paternity of the cell growth curve. Based on the growth curve data of the primary cells, the population doubling time (PDT) was calculated to be approximately 26.5±0.7 hours (Fig. 4).

### Cell authentication by STR:

**Fig. 5:**
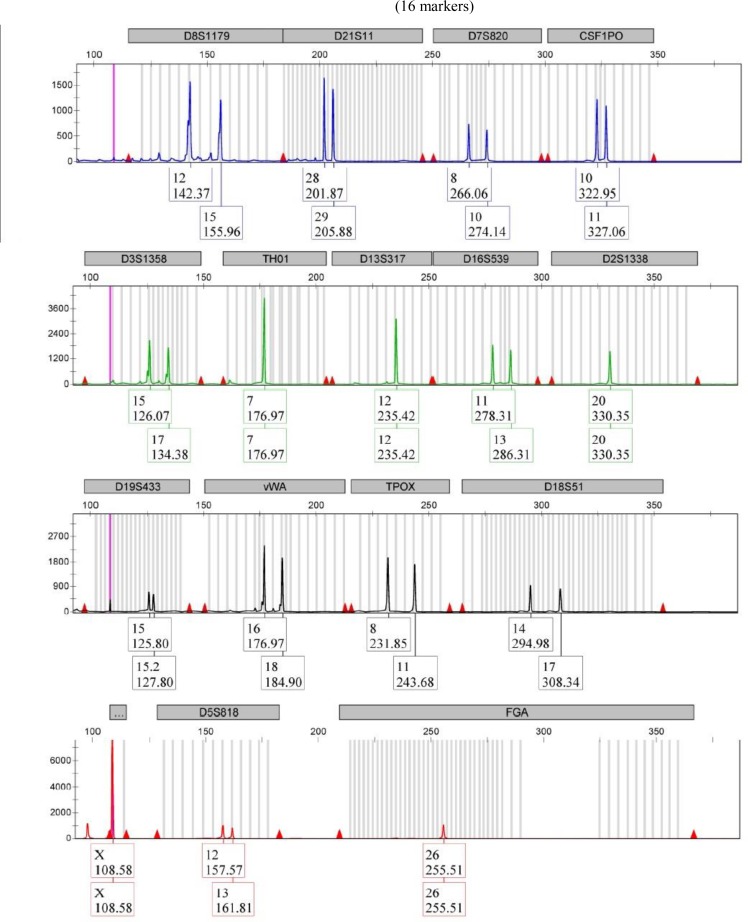
STR profile for authentication of OSCC cell line

STR profiling helps in the detection of misidentified, cross-contaminated or genetically drifted cells. STR profiling is a quick, reproducible and standardized PCR-based technique for authentication of human cell lines. All the samples were profiled individually by STR markers, and no misidentified or cross-contaminated cells were detected (Fig. 5).

### Flow cytometry analysis:

EpCAM (CD326) is a Ca^2+^ independent adhesion molecule, generally expressed on the basolateral surface of epithelial and carcinoma cells in OSCC. Here, the established primary oral cancer cells were characterized by CD marker expression using flow cytometry, and the results revealed high levels of CD326.

**Fig. 6:**
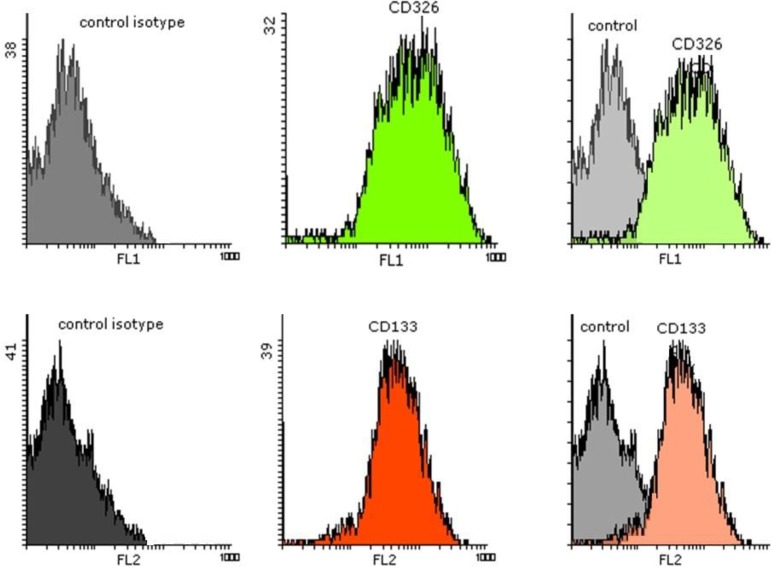
Flow cytometry analysis disclosed the positive expression of primary oral cancer cells for CD326 (green histogram), and CD133 markers (red histogram)

CD133 (prominin-1) is presently considered a useful biomarker for prognosis and detection of cancer stem cell in a variety of human cancers, including oral cancer. The CD133 positive cells were examined by flow cytometry, and the results showed that the cells were positive for this marker (Fig. 6).

## DISCUSSION

Typically, two-dimensional (2D) monolayer cell culture models are the most common cell culture systems. But recently, three-dimensional (3D) culture systems are being used for anticancer drug discovery, cancer cell biology, cancer stem cell study and other cell-based analyses. It seems that 3D cell culture systems are able to fill the gap between 2D cell culture systems and body systems in which cell-cell and cell-substrate interactions retain the in-vivo morphology in 3D cultures.

In this line, spheroid culture models with their 3D nature are also used as excellent systems in cancer cell studies.

Although 3D cell cultures have some advantages over 2D techniques, monolayer cell cultures are still used for most studies, due to their well-establishment and easier cell observation and measurement [11,12]. Current studies are focused on producing primary oral squamous cancer cells to facilitate further researches on therapeutic approaches to improve patients’ survival [13]. Human cancer cell lines in primary cultures are commonly used in cancer researches when formalin-fixed, paraffin-embedded specimens do not provide an appropriate material for these studies. During recent years, several cancer cell lines have been established from human cancers of the whole body [14, 15]. However, a low success rate in the establishment of SCC cell lines has been reported in the literature [16–19]. A major concern about these cell lines is that their genetic features may dramatically change after a long period of time or a huge number of passages. [20, 21]. Therefore, it is recommended to use newly-established primary cells with a low passage number for cancer studies. Several studies have demonstrated that geographical diversities have great impacts on genetic abnormalities in human cancer. In other words, it seems that carcinogenic processes are greatly environmental-dependent [22]. Recently, a novel consideration about treatment approaches entitled as “Personalized Medicine” has been evolved, which was initially triggered by molecular profiling of cancers. This hypothesis emphasized the necessity of a well-descriptive material to identify genetic and biochemical characteristics of various patients with similar cancer, which may be provided by primary cultured cells [23]. Here, we decided to establish primary oral cancer cells specified for the Iranian race, according to the idea that the development of human cancers is linked to racial and environmental factors. The five primary cell cultures were developed from untreated primary tumors from an Iranian genetic background. The main reason for low success rate of establishment of primary cells, especially cells originated from epithelial tissues, is contamination with non-tumoral cells, including normal fibroblasts and carcinoma-associated fibroblasts (CAFs), and some other cells such as endothelial cells and inflammatory cells, as well as infection with fungal hyphae, bacteria, and mycoplasma [7]. In the current study, the five primary oral cancer cells were well-established from more than 10 tumoral specimens. Although it seems that tumoral complexity and heterogeneity have been well-introduced by primary cancer cells, it is technically difficult to establish patient-derived low passage cancer cell lines from fresh tumors in vitro. This is due to the difficulties of isolating profound malignant clones, as the size and number of fresh tumor specimens are limited due to natural heterogeneity and multiclonality of human cancers [24, 25]. Another problem raised by this study was difficulties in receiving fresh, micro-dissected malignant specimens. Any delay in receiving the resected tumor could increase the risk of contamination. Therefore, tumoral specimens received after 17 minutes of excision time were excluded from the study, in order to avoid further complications. Standard culture media containing antibiotics and antifungal substances were used for transferring tumor specimens to the laboratory, immediately after resection. In terms of culturing method, the explant technique is one of the methods used in cell isolation and in-vitro cell dissemination. In this technique, the tissue size was small enough to allow the diffusion of nutrients and gases [26]. The migration ability of the cells is an important factor in successful primary explant culture [27]. Another technique for cell isolation is the use of enzymatic systems including trypsin and/or collagenases for tissue digestion. The digestion outcome, defined as the efficacy, viability, yield and toxicity, is affected by the type of the enzyme used in this method [28]. In one study, no significant differences in cell isolation yield were reported between explant culture method and enzymatic method. Their findings also revealed that explant techniques are noninvasive and relatively easy [29].

In the present study, enzymatic and explant methods were used to isolate oral cancer cells. In both methods, epithelial cells were successfully isolated from the tissue. However, each method has some advantages and disadvantages. In the enzymatic method, epithelial cells were observed 12 hours after the primary culture, while in the explant method, the first sign of cell migration out of the tissue appeared after 48 hours. Hence, the enzymatic method could be a faster and more efficient approach in terms of high yield for isolating primary carcinoma cells.

## CONCLUSION

We established five human primary oral cancer cells from an Iranian population with different purity, which would provide a suitable tool for studies on cancer focusing on personalized treatment plans. These human primary cells could be employed to evaluate anticancer drugs for OSCC, to develop future research on oral cancer and to revolutionize conventional oral cancer therapy.
